# Task-switching mechanisms under methamphetamine cravings: sex differences in cued and voluntary task-switching

**DOI:** 10.3389/fnins.2024.1462157

**Published:** 2024-10-30

**Authors:** Huan Jiang, BinJie Yang, HanZhi Huang, Dong Zhao, HuiLing Li, ZhiYing Chen, Shengyi Jin, Qiang Zhou

**Affiliations:** ^1^Department of Psychology, Wenzhou Medical University, Wenzhou, China; ^2^Lishui Second Hospital Affiliated to Wenzhou Medical University, Lishui, China; ^3^Shanghai Key Laboratory of Brain Functional Genomics, School of Psychology and Cognitive Science, Ministry of Education, East China Normal University, Shanghai, China; ^4^Zhejiang Moganshan Female Drug Detoxification Center, Huzhou, China; ^5^Wenzhou Ouhai District Anti-drug Committee Office, Wenzhou, China

**Keywords:** methamphetamine, drug cravings, sex differences, task-switching, brain mechanisms

## Abstract

**Introduction:**

This study explored the effects of task-switching type and sex on the task-switching ability of methamphetamine abstainers, as well as the differences in brain mechanisms under drug cravings under drug cravings using near-infrared spectroscopy.

**Methods:**

Craving-inducing videos were used to arouse 20 methamphetamine abstainers (including 10 men), whose switching ability was then assessed using voluntary and cued task-switching exercises.

**Results:**

During task-switching under methamphetamine cravings, the activation of the premotor cortex (PMC), supplementary motor area (SMA), frontal eye field (FEF), and dorsolateral prefrontal cortex (DLPFC) in women was significantly stronger than in men, while the activation of FEF in men was significantly stronger than in women. Voluntary task-switching induced stronger FEF activation than cued task-switching. During the latter, women exhibited stronger activation in the anterior prefrontal cortex (aPFC) than men.

**Discussion:**

Both men and women showed brain lateralization during task-switching under methamphetamine cravings. Men tended to adopt proactive control and use a top-down dominant strategy to start a new task. Women, however, tend to use a bottom-up strategy focusing on inhibiting old tasks and emotional switching. Moreover, in cued task-switching, the result shows women paid more attention to emotional processing than did men, which suggests that different task-switching training programs should be developed according to sex.

## 1 Introduction

Drug addiction is a persistent healthcare and social challenge, following the problem of opioid addiction, the problems posed by methamphetamine use have become a new challenge (Paulus and Stewart, 2020; Kevil et al., 2019). Methamphetamine use has risen rapidly over the past decade or so to become one of the most widely abused drugs in the world (Ben-Yehuda and Siecke, 2018; Stoneberg et al., 2018). In China, methamphetamine is also one of the most commonly abused drug, accounting for 55.2% of the drug abuse population (Liu and McNally, 2021). Although abstainers receive systemic treatment for addiction, the high relapse rate remains a common problem in the field of drug rehabilitation (Tatari et al., 2021). In a 10-year prospective follow-up research (Xie et al., 2005), all the clients relapsed in the first year or during the whole period. We performed a meta-analysis of the relevant literature published between 1975 and October 2022 (see all the details and references in Supplementary Appendix 1), and found that the overall relapse rate for the top five drugs was 43.4%, with methamphetamine having the highest relapse rate of 59.7%. Many studies have found that even though many users seek treatment, most of them still relapse (Garfield et al., 2021; Brecht and Herbeck, 2014; McKetin et al., 2012). Therefore, the underlying mechanism of methamphetamine cravings and the prevention and treatment of relapse should receive more attention in the addiction research field (Tiffany, 1999; Chang et al., 2007).

The main trigger for methamphetamine relapse is drug craving (Chen et al., 2019), which is a strong desire to consume again a previously experienced psychoactive substance. According to the incentive-sensitization theory, changing addicts’ automatic attentional bias toward drugs is a fundamental goal of reducing relapse (Muraven and Shmueli, 2006; Parvaz et al., 2017). In addition to this, biochemical and psychosocial vulnerability affects anger rumination and cognitive flexibility, which may place methamphetamine withdrawal that has undergone systematic abstinence at greater risk for relapse or recidivism upon release from prison (Herschl et al., 2012).

Task-switching is the core mechanism of cognitive control, the ability to flexibly switch tasks when the goal is shifted (Leite et al., 2013; Luc et al., 2019). According to different cognitive processing styles, task-switching can be divided into cued and voluntary. The former adopts a bottom-up cognitive approach, requiring subjects to perform corresponding tasks according to random cues (Meiran et al., 2000). The latter is closer to the reality of life. Through top-down cognitive processing, subjects can choose the task to be performed and determine the number of executions and the switching time (Arrington and Logan, 2004). Task-switching measures typically require participants to perform two different types of cognitive tasks. Depending on whether the current task is the same as or different from the previous task, it is defined as a repeat task or a switch task; switch tasks have longer reaction times and lower accuracy rates than repeat tasks, known as switch costs (Monsell, 2003).

In Van der Plas et al. (2009) study, methamphetamine addicts performed poorly in task-switching and showed impairments in complex decision-making, working memory, and cognitive flexibility. In addition, impairment of the executive function, including cognitive flexibility, in this population, might be an inducing factor for initial use or relapse after rehabilitation (Miller, 1991). A recent study using the cued task-switching paradigm found that methamphetamine addicts showed reduced proactive control and mobilized additional reactive control to compensate to some extent, indicating that they have an impaired ability of proactive control during task-switching (Su and Zheng, 2023). Additionally, increasingly more researchers have paid attention to sex-related brain lateralization effects and corresponding cognitive neurobehavioral differences in different cognitive tasks (Eliot, 2011). In women, visual task-switching is controlled by neuronal networks in the dorsolateral prefrontal cortex (DLPFC), inferior parietal region, secondary visual areas in both hemispheres, and cerebellar cortex, while in men, in addition to these areas, visual task-switching also involves the right supplementary motor area, right insula, and left thalamus (Kuptsova et al., 2015). Men show greater flexibility in visuospatial perception (Tschernegg et al., 2017). At present, however, it is not clear that there are sex differences intask-switching types.

This study aims to use functional near-infrared spectroscopy (fNIRS) to investigate brain activation differences between male and female participants during voluntary and cued task-switching under methamphetamine cravings. The specific objectives are: (1) to reveal the neurophysiological mechanisms of task-switching under methamphetamine cravings; and (2) to compare the sex differences in the two types of task-switching.

## 2 Materials and methods

### 2.1 Participants

Sample size calculations were performed using G*Power software. Based on the expected effect size f of 0.5, the significance level set at 0.05, and the power of the target test set at 0.80, we calculated the required sample size to be 12.

A total of 20 methamphetamine quitters, including 10 males, were recruited from Wenzhou Huanglong Compulsory Isolation Drug Rehabilitation Center (men’s rehabilitation center) and Zhejiang Moganshan Female Drug Detoxification Center. The Participants were aged (32.35 ± 5.73) years, had been using drugs for (6.90 ± 4.49) years, and had a drug-using background of (15.90 ± 3.39) years, were right-handed, had normal or corrected vision, and mastered the judgment of numerical parity and size.

The inclusion criteria were (a) meeting the addiction criteria of the Chinese Classification of Mental Disorders, Third Edition (CCMD-3) (Chen, 2002), (b) at least one year of methamphetamine use, and (c) 1 to 2 years of methamphetamine abstinence. Exclusion criteria included (a) history of psychiatric and neurologic disorders, (b) use of psychotropic or therapeutic medications, and (c) history of co-morbidities.

The experiment was approved by the Ethics Committee of Wenzhou Medical University (2022-016), and participants signed an informed consent form before the experiment.

### 2.2. Materials

#### 2.2.1 Video material

The thirst video was provided by Yuan Ti Fei’s research team (Zhao et al., 2021), the video content is a real methamphetamine smoking scene provided by the informant, the smoking methods are hot sucking and bottle water soluble, the length of the video is 270s.

#### 2.2.2 Craving scale

The 13-item Desire for Drug Questionnaire (DDQ) developed by Franken et al. (2002) is a frequently used instrument for measuring the level of instant craving for drugs (Supplementary Appendix 2). The DDQ measures three factors: desire and intention, negative reinforcement, and control, and demonstrates high reliability and concurrent validity for patients receiving treatment for drug dependency, and thus it can be employed in both clinical and research fields. The higher the total score, the higher the participant’s craving for drugs. In this study, we used the Chinese version of the DDQ (Li et al., 2024), whose reliability and validity have been validated in previous studies (Yen et al., 2016).

#### 2.2.3 Visual simulation scoring method

A visual simulation scale is used to quantify how much a participant craves methamphetamine at a given moment. It consists of an approximately 10-cm-longmoving scale with 10 scales on one side and “0” and “10” scales at each end, with 0 indicating no craving at all and 10 representing the most intense craving that is difficult to tolerate.

### 2.3 Procedure

**FIGURE 1:**
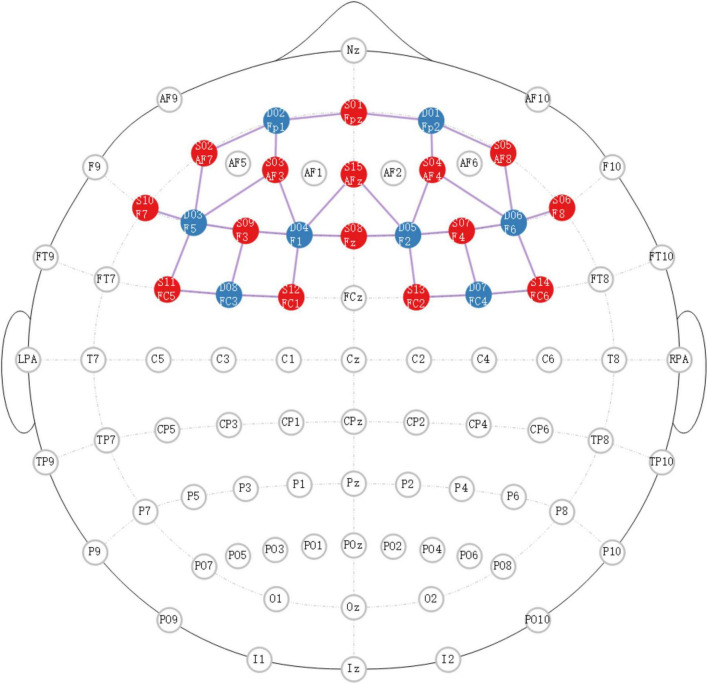
The specific localization of source detector. Red dots represent the transmitting electrodes, blue dots represent the receiving electrodes, purple dots represent the channels.

The experiments were presented using E-prime 3.0 and monitored by NIRScout (NIRX Medical Technologies LLC, USA). The primary region of interest was situated within the prefrontal cortex area, and the specific localization of the source detector is depicted in Figure 1.

**FIGURE 2:**
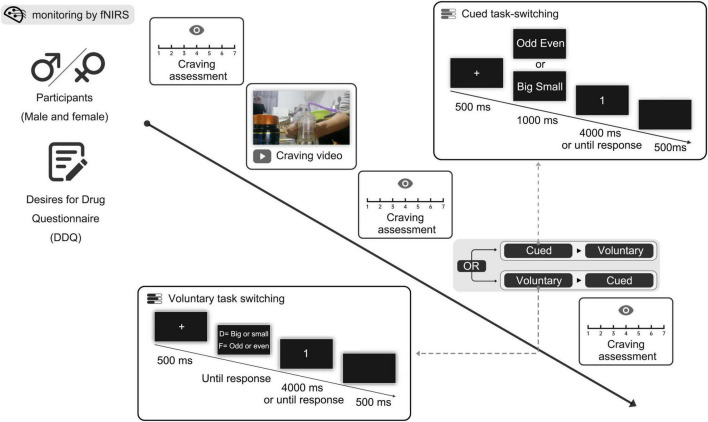
Illustration of the procedure.

Before the whole experiment, background craving (participants’ self-ratings of drug-crave in the absence of any drug-related stimuli) was measured. Participants regulated their breathing and maintained a tranquil state for 1 minute before viewing the craving-inducing video, after which they provided a second subjective methamphetamine craving score. A counterbalanced experimental design encompassing both voluntary and cued task-switching paradigms was implemented, with a practice phase (comprising 16 trials) and a formal phase (comprising 64 trials). After completion of the corresponding task, participants were required to provide a third subjective assessment of their methamphetamine craving level. An illustration of the experimental procedure is presented in Figure 2.

#### 2.3.1 Voluntary task switching

Initially, a 500ms fixation point was displayed, succeeded by a task selection screen offering options for “size” and “parity.” Participants were instructed to independently press the keys “D” or “F” in a randomized manner to select and assess the size or parity of the presented stimuli (< 4000 ms. The stimuli of the task were the digits 1, 2, 3, 4, 6, 7, 8, and 9 presented in black on a white background). Following each trial, a 500ms blank interval appears before the commencement of the subsequent trial. Participants underwent a practice session before the formal experiment. In order to ensure that participants fully understand the requirements of the experiment, participants will be required to meet the following criteria during the self-directed task-transition exercise phase before entering the formal experiment: accuracy rate ≥ 80%, 30% ≤ task switching rate (equal to the number of switches divided by the total number of tasks) ≤ 70%, 30% ≤ task selection rate (equal to the number of times two different tasks were completed divided by the total number of tasks) ≤ 70%.

#### 2.3.2 Explicitly cued task-switching

The procedure mirrors that of the voluntary task-switching, with the distinction that, rather than a task selection screen, the fixation point was followed conspicuously by either a “size” or “parity” task cue.

### 2.4 Data analytic plan

SPSS 23.0 was used for statistical analysis of the effectiveness and persistence of craving arousal. A *t-*test was conducted to assess potential sex disparities in craving levels and to ascertain if these levels exhibited any changes before and after viewing the video. Furthermore, a two-factor repeated measures ANOVA, incorporating a 2 (sex: women/men) × 2 (task-switching type: voluntary/cued) design, was carried out to investigate the influence of methamphetamine craving and task-switching type on the switching cost.

Nirslab was used to set the fNIRS signal correlation events with “discontinuity” and “spike artifact” to eliminate motion artifacts caused by head movement and electrode displacement, and band-pass filtering with a high cutoff frequency (0.2) and a low cutoff frequency (0.01) to eliminate the effects of instrumentation, drift, and other noise. The modified Beer-Lambert law was applied to extract oxy-Hb and deoxy-Hb. fNIRS recordings were initiated in the resting state and recorded and analyzed for 2 min in the resting state; in both autonomous and cued task transitions, responses were recorded and analyzed for 8s after the appearance of the digital stimulus for each transition.

The segmented fNIRS signals were examined separately in the data analysis module using statistical parametric plot software. Although both oxy-Hb and deoxy-Hb reflect cerebral blood flow in fNIRS, oxy-Hb is more sensitive than deoxy-Hb (Hu, 2018). Therefore, only oxy-Hb was further analyzed: based on the assumption that the hemodynamic response function peaks at 5 s (Boynton et al., 2012), the mean of the entire timeline was used as a baseline (Oku et al., 2022), and the mean change in oxy-Hb per task-switching event was calculated for both voluntary and cued conditions.

Analysis of the fNIRS data was based on a general linear model (Y = Xβ + ε), where β denotes the strength of the relationship with y, suggesting the degree of cortical activation (Perpetuini et al., 2019). The data were entered into a generalized linear model to obtain β-values for oxy-Hb, and finally, a two-factor ANOVA was performed by SPSS 23.0 to analyze the differences in 32-channel activation between autonomous and cued task-switching by sex. In addition, the results of statistical tests between multiple channels were corrected using the false discovery rate method (FDR).

## 3 Result

### 3.1 Methamphetamine craving arousal

Paired-sample t-tests found that the second score (*M* = 2.35, SD = 3.33) was significantly higher than the first (*M* = 0, SD = 0, *t (19)* = −3.16, *p* = 0.005, Cohen’s d = 0.998)which indicates craving arousal was effective, and the difference between the second (*M* = 2.35, SD = 3.33) and the third (*M* = 1.50, SD = 2.78) was not significant (*t (19)* = 1.35, *p* = 0.193, Cohen’s d = 0.277), suggesting that craving was sustained during the secondary craving measure.

### 3.2 Behavioral experiment

An ANOVA on switching costs found a significant main effect of switching type (*F*_(1,18)_ = 7.034, *p* = 0.016, *η_*p*_^2^* = 0.281), and the switching costs of voluntary switching (*M* = 132.296, SD = 115.495) were greater than cued switching (*M* = 61.888, SD = 106.579).

The main effect of sex was not significant (*F*_(1,18)_ = 0.154, *p* = 0.699, *η_*p*_^2^* = 0.008), but the interaction between sex and switching type was significant (*F*_(1, 18)_ = 17.520, *p* < 0.001, *η_*p*_^2^* = 0.493). Further simple effects analyses found that in the voluntary switching condition, the switching cost was higher for women (*M* = 194.674, SD = 30.909) than for men (*M* = 69.919, SD = 30.909), whereas in the cued-switching condition it was the men who had a higher switching cost (*M* = 110.630, SD = 30.909) than the women (*M* = 13.145, SD = 30.909). And among women participants, the switching cost of voluntary switching (*M* = 194.674, SD = 30.909) was significantly higher than cued switching (*M* = 13.145, SD = 30.909).

There were no significant effects for the other outcomes. The results are summarized in Table 1.

**TABLE 1:** Behavioral data of participants of different sex in task-switching experiments.

Behavioral indicators	Sex	M	SD	Max	Min
Switch rates _voluntary_	Women	0.45	0.16	0.73	0.19
	Men	0.47	0.09	0.59	0.29
Switch RT _voluntary_	Women	1000.86	272.56	1609.03	632.43
	Men	850.26	160.59	1236.58	684.80
ACC _voluntary_	Women	0.89	0.11	0.97	0.73
	Men	0.90	0.04	0.95	0.46
Repetition RT _voluntary_	Women	806.19	196.21	1291.50	579.00
	Men	784.53	161.08	1214.05	625.76
Switch RT _cued_	Women	872.26	317.27	1374.65	520.63
	Men	824.60	150.48	1007.88	570.37
ACC _cued_	Women	0.93	0.04	1.00	0.86
	Men	0.93	0.09	1.00	0.70
Repetition _cued_	Women	866.11	350.31	1383.69	451.75
	Men	777.03	193.46	1172.18	471.68

### 3.3 fNIRS experiment

**FIGURE 3:**
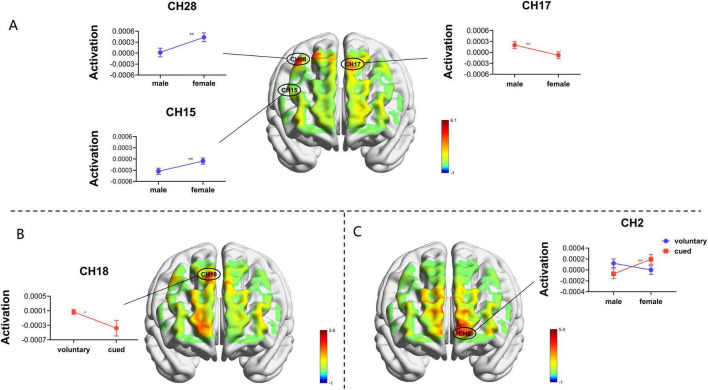
Neural correlates of task-switching types and sex. F-test maps of brain activation generated based on ANOVAs. **(A)** Brain activation maps for sex differences under task-switching;**(B)** Differential activation maps for task switching types; **(C)** Sex-differentiated brain activation maps in cue task-switching. (**p < 0.05, *0.05 < *p* < 0.01).

In Channel 18, there was a marginally significant main effect of switching type (*F*_(1, 18)_ = 3.555, *p* = 0.076, *η_*p*_^2^* = 0.165), As shown in Figure 3B, with a greater β-values for voluntary switching than for cued switching; increased activation of the right FEF.

In Channel 17 (*F*_(1, 18)_ = 4.535, *p*_*corrected*_ = 0.047, *η_*p*_^2^* = 0.201), the main effect of sex was significant, men had larger β-values than women; increased activation of the left FEF. Channel 15 (*F*_(1, 18)_ = 5.466, *p*_*corrected*_ = 0.047, *η_*p*_^2^* = 0.233), and Channel 28 (*F*_(1, 18)_ = 6.076, *p_*corrected*_* = 0.047, *η_*p*_^2^* = 0.252) had a significant main effect of sex, with women having larger β-values than men; increased activation of the right PMC, right SMA, and right DLPFC. As presented in Figure 3A.

In Channel 2 (*F*_(1,18)_ = 5.383, *p* = 0.032, *η_*p*_^2^* = 0.230) the interaction between sex and switch type reached significance, and a simple effects analysis revealed that in the cue-switching condition, as depicted in Figure 3C, women’s β-values were greater than men’s; with increased activation in the aPFC.

There were no significant effects for the other Channels. The results are summarized in Supplementary Appendix 3.

## 4 Discussion

At the behavioral level, our study has found that cued task-switching is less costly than the voluntary one under methamphetamine cravings; that is, the task-switching driven by exogenous cues is easier than that by proactive control, which may be related to the impairment of proactive control caused by methamphetamine addiction (Salo et al., 2011). Previous research have found that deficiencies in proactive control lead patients to use methamphetamine despite victimization (Paulus and Stewart, 2020; Potvin et al., 2018), so the development of proactive control is critical to managing methamphetamine use and withdrawal (Opitz et al., 2023). Interestingly, the switching cost of voluntary switching was higher than cued switching among women participants. There exists an interpretation that methamphetamine abstainers (women in particular in our study because they are better at cued switch) may develop attentional bias triggered by drug-related cues and enhance the bottom-up neural processing (Ihssen et al., 2017; Nestor et al., 2011), but the top-down control of attention has not benefited from this (Leganes-Fonteneau et al., 2019).

We also found sex differences in different styles of task-switching, with behavioral results showing advantages of cued switching in women and of voluntary switching in men. Although no significant effect of sex has been found in previous studies that did not specifically distinguish the switching types (Hirsch et al., 2019), Solianik et al. (2016) found that in unpredictable task-switching (similar to voluntary switching), the coefficient of variation within the male group was significantly lower than that of females, implying that males as a group outperformed females in task-switching without relevant cues. In addition, Lui and Wong (2020) argued that multitasking ability can be categorized into multiple facets, and there are differences in the specific facets in which females and males excel. Among them, females outperform males in task information extraction and maintenance, while males have better response selection abilities in the final choice session, making males more adept at making more than one choice quickly in a parallel dual task. This explanation is consistent with our behavioral results that women can process information faster than men in cued task-switching where the switching information is explicit and responds quickly accordingly. Similarly, while men may not be as good as women at switching information processing and maintenance, faster final choice make them better at voluntary task-switching.

At the neural level, we found the activation of FEF much stronger in voluntary than in cued task-switching, the FEF is a critical region for the deployment of overt and covert spatial attention, which plays a role in eye movement production (Maljkovic and Nakayama, 2000; Muggleton et al., 2010), it rapidly switches activation modes to accommodate goal-directed and stimulus-driven attentional conditions (Asplund et al., 2010). In voluntary task switching, due to the pre-preparation before each switch, timely and highly focused attentional resources are devoted to observing the speed of attentional switching (Gruber et al., 2006). It can be inferred that voluntary task switching is better at detecting changes in the external environment than cue-driven task switching. Although task-switching by proactive control is more difficult than that driven by cues under methamphetamine cravings, voluntary task-switching is more sensitive to the changes of external stimuli, so it is better suited to the actual situation where abstainers need to consciously resist temptations after returning to society. Therefore, this ability needs to be improved, especially during drug-craving states.

Further, we also found that there were sex-related brain lateralization differences in the PMC, SMA, FEF, and DLPFC under methamphetamine cravings. The activated brain areas tended to be located in men’s left side and women’s right side. Previous studies have shown that the dorsal premotor cortex plays a crucial role in visually guided goal-directed motor behavior (Kim, 2011; Nakayama et al., 2022), which may be related to perceptual transformation of target stimuli during task execution. DLPFC plays a role in working memory, goal-driven attention, task-switching, planning, problem-solving, and novelty-seeking (Aboulafia-Brakha et al., 2016; Jones and Graff-Radford, 2021), which may help individuals perform task-switching under the emotional state of drug craving. However, the specific functions of the left (l) and right (r) sides of these two brain regions are inconsistent. The rDLPFC is mainly involved in inhibition, which simply indicates “I won’t do it” (Friehs et al., 2020); instead, lDLPFC dominates the excitatory action, that is, “I will do it.” In our study, men’s stronger activation on the lDLPFC may reflect their stronger active control in the task and therefore better performance in voluntary task-switching. In a rules-based study on visual motion and visual object mapping, the lDLPFC was more involved in the top-down process of action preparation than the rDLPFC (Nakayama et al., 2022). This may suggest that in task-switching, the left lateralized activation in men is a strategy that favors proactive control and helps initiate new tasks, while the right lateralized activation in women is a strategy by which they tend to be instructed and inhibit old tasks.

PFC also revealed the sex differences, in cued task-switching, the activation in the PFC was stronger in women than in men. Previous studies have shown that, under significant time pressure and rapidly changing environmental requirements, the function of rapid switching of different emotion control strategies relies on the PFC (Koch et al., 2018). Drug craving is an emotional-motivational state of subjective desire for drug use (Potgieter et al., 1999; Merikle, 1999). Craving for drugs was a significant predictor of emotional manipulation ability (Khedr et al., 2023). Therefore, in cued task-switching, women might be more inclined than men to switch tasks when they are resisting the emotional state of drug cravings, which may be related to their better emotional expression in emotional control. Neuropsychological studies have found that, during emotional regulation, PFC shows higher activation related to cognitive processing in men and higher activation related to emotional processing in women. In particular, women presented stronger activation in the left medial orbitofrontal gyrus in processing negative emotions compared with men, with better task performance (Mak et al., 2009).

In summary, men and women showed lateralized brain mechanisms underlying task-switching during methamphetamine cravings. Men were better at proactive control, while women tended to be passively driven. In cued task-switching, women paid more attention to emotional processing. This study focused on abstainers’ “autonomy” to divert their attention under a state of craving and expanded the application of voluntary task-switching to methamphetamine abstainers. The results have significance for the treatment of methamphetamine addicts, where attention diversion is a useful ability. However, there were some shortcomings. First, recruiting abstainers who have returned to society to conduct the study would have been of more significance for drug relapse management. However, because of practical obstacles of organization and management, the subjects of this study were all addicts in drug rehabilitation centers, and the sample size was limited, so in future experimental studies, more places can be considered to recruit forced ex-addicts who have returned to the society within a certain time frame. Second, the “autonomy” in the voluntary task-switching paradigm used in this study was guided by instructions, and it was difficult to ensure that all subjects had made their own choices in all trials. Future studies can ensure a higher degree of actual autonomy for participants. Third, future research could attempt to combine the task-switching paradigm with techniques that have been shown to work well with addicted patients, such as transcranial magnetic stimulation (TMS, Martinotti et al., 2022), transcranial direct current stimulation (tDCS, Martinotti et al., 2019), and cognitive behavioral therapy (CBT, McHugh et al., 2010).

## Data Availability

The datasets presented in this study can be found in online repositories. The names of the repository/repositories and accession number(s) can be found in the article/Supplementary material.
