# Supplementary material for: Task-switching mechanisms under methamphetamine cravings: sex differences in cued and voluntary task-switching
Source: Front Neurosci. 2024 Oct 30;18:1462157. doi: 10.3389/fnins.2024.1462157 (PMC11557557; doi:10.3389/fnins.2024.1462157)
Supplement: Supplementary file 1 [file Data_Sheet_1.pdf]

Meta analysis

A total of 3156 articles were searched in the SCI-HUB, PUBMED and PsycInfo databases using the search terms "Cannabis, Opioids, Amphetamines, Cocaine, Ecstasy" for drugs and "Relapse" for relapse, and the publication period was from 1975 to October 7, 2022. The following criteria were used for screening: (1) relapse rates were reported; (2) relapse rates were reported within one year of the participant's return to the community after being detoxified by a detoxification facility; (3) the relapse rate was only specific to a particular type of drug; and (4) the participant did not suffer from schizophrenia. Our final analysis obtain 31 articles, two of which had two sub-studies, resulting in a total of 33 effect sizes.

In order to generalize the results of the meta-analysis (Borenstein et al., 2009), statistical analyses were conducted based on a random effects model. Comprehenesive Meta-Analysis 2.2 was adopted to conduct the meta-analysis. First, heterogeneity was tested by q-test, and the result was significant ($P < 0.001$), indicating that the effect sizes of the original study were not similar; then, according to the display of the funnel plot (shown in Figure 1-1), the points on the left and right sides of the symmetry axis were not uniform, suggesting that there might be a possible publication bias; and further loss of safety coefficient test was performed along with the Egger 's test in order to more accurately test the possibility of publication bias. According to the Egger 's test, $P > 0.05$, indicating the existence of publication bias; whereas, according to the loss of safety coefficient test, the inclusion of 9,411 documents was required to make the effect size insignificant, indicating the absence of publication bias (e.g., Table 1-1). In the event of inconsistency between the results of the two publication bias tests, the trim-and-fill method proposed by Duval and Tweedie was further used to further examine the effect of publication bias on the results of the meta-analysis (Duval et al., 2000), and it was found that the overall effect obtained using the random effects model was still significant after trimming and filling the research literature.

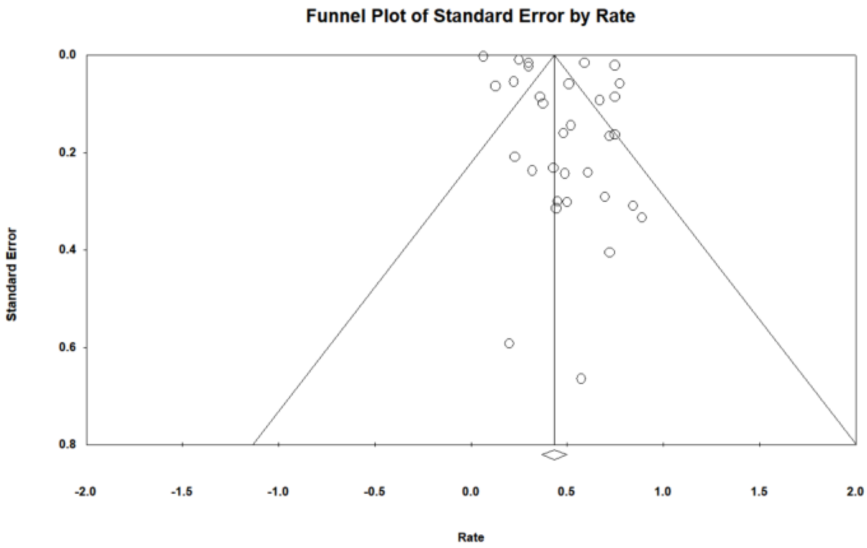

Figure 1-1 Funnel plot

(Note: The vertical axis is the log standard error of the effect size, the horizontal axis is the effect size, the inner funnel is the confidence interval, and the center axis is the combined effect size.)

Table 1-1 Results of publication bias tests

Rosenthal's N	Egger'intercept	SE	LL	UL	p
9411	5.65	1.64	2.30	8.99	<0.05

(Note: LL and UL denote the upper and lower bounds of Egger's intercept at the 95% confidence interval, respectively.)

The final result showed that the total relapse rate was 0.434. Among them, amphetamines (all methamphetamine) was 0.597; heroin was 0.498; cocaine was 0.438; and marijuana was 0.218. It can be seen that nearly half of the abstinent persons relapsed within one year after returning to the society, and the relapse rate of methamphetamine was the highest, as shown in Table 1-2 for details.

Table 1-2 Results of random effects model analysis

k	r	LL	UL	Z	p
33	0.434	0.37	0.50	13.05	<0.05

(Note: N represents the sample size, K represents the number of studies, and LL and UL represent the upper and lower bounds of the interval of R in the 95% confidence interval, respectively).

Reference

*Abdoli, N., Farnia, V., Radmehr, F., Alikhani, M., Moradinazar, M., Khodamoradi, M., ... & Davarinejad, O. (2021). The effect of self-compassion training on craving and self-efficacy in female patients with methamphetamine dependence: a one-year follow-up. Journal of Substance Use, 26(5), 491-496. https://doi.org/10.1080/14659891.2020.1851406

*Aharonovich, E., Liu, X., Samet, S., Nunes, E., Waxman, R., & Hasin, D. (2005). Postdischarge cannabis use and its relationship to cocaine, alcohol, and heroin use: a prospective study. The American journal of psychiatry, 162(8), 1507–1514. https://doi.org/10.1176/appi.ajp.162.8.1507

*Aryan, N., Banafshe, H. R., Farnia, V., Shakeri, J., Alikhani, M., Rahimi, H., Sehat, M., Mamsharifi, P., Ghaderi, A., & Omidi, A. (2020). The therapeutic effects of methylphenidate and matrix-methylphenidate on addiction severity, craving, relapse and mental health in the methamphetamine use disorder. Substance abuse treatment, prevention, and policy, 15(1), 72. https://doi.org/10.1186/s13011-020-00317-y

*Arendt, M., Rosenberg, R., Foldager, L., Sher, L., & Munk-Jørgensen, P. (2007). Withdrawal symptoms do not predict relapse among subjects treated for cannabis dependence. The American journal on addictions, 16(6), 461–467. https://doi.org/10.1080/10550490701640985

*Back, S. E., Hartwell, K., DeSantis, S. M., Saladin, M., McRae-Clark, A. L., Price, K. L., Moran-Santa Maria, M. M., Baker, N. L., Spratt, E., Kreek, M. J., & Brady, K. T. (2010). Reactivity to laboratory stress provocation predicts relapse to cocaine. Drug and alcohol dependence, 106(1), 21–27. https://doi.org/10.1016/j.drugalcdep.2009.07.016

*Boden, M. T., McKay, J. R., Long, W. R., & Bonn-Miller, M. O. (2013). The effects of cannabis use expectancies on self-initiated cannabis cessation. Addiction (Abingdon, England), 108(9), 1649–1657. https://doi.org/10.1111/add.12233

*Bonn-Miller, M. O., & Moos, R. H. (2009). Marijuana discontinuation, anxiety symptoms, and relapse to marijuana. Addictive behaviors, 34(9), 782–785. https://doi.org/10.1016/j.addbeh.2009.04.009

*Brecht, M. L., & Herbeck, D. (2014). Time to relapse following treatment for methamphetamine use: a long-term perspective on patterns and predictors. Drug and alcohol dependence, 139, 18–25. https://doi.org/10.1016/j.drugalcdep.2014.02.702

*Brecht, M. L., von Mayrhauser, C., & Anglin, M. D. (2000). Predictors of relapse after treatment for methamphetamine use. Journal of psychoactive drugs, 32(2), 211–220. https://doi.org/10.1080/02791072.2000.10400231

*Buydens-Branchey, L., & Branchey, M. (2003). Association between low plasma levels of cholesterol and relapse in cocaine addicts. Psychosomatic medicine, 65(1), 86–91. https://doi.org/10.1097/01.psy.0000039754.23250.ee

*Abdoli, N., Farnia, V., Radmehr, F., Alikhani, M., Moradinazar, M., Khodamoradi, M., ... & Davarinejad, O. (2021). The effect of self-compassion training on craving and self-efficacy in female patients with methamphetamine dependence: a one-year follow-up. Journal of Substance Use, 26(5), 491-

496. https://doi.org/10.1080/14659891.2020.1851406
*Aharonovich, E., Liu, X., Samet, S., Nunes, E., Waxman, R., & Hasin, D. (2005). Postdischarge cannabis use and its relationship to cocaine, alcohol, and heroin use: a prospective study. The American journal of psychiatry, 162(8), 1507–1514. https://doi.org/10.1176/appi.ajp.162.8.1507
*Aryan, N., Banafshe, H. R., Farnia, V., Shakeri, J., Alikhani, M., Rahimi, H., Sehat, M., Mamsharifi, P., Ghaderi, A., & Omidi, A. (2020). The therapeutic effects of methylphenidate and matrix-methylphenidate on addiction severity, craving, relapse and mental health in the methamphetamine use disorder. Substance abuse treatment, prevention, and policy, 15(1), 72. https://doi.org/10.1186/s13011-020-00317-y
*Arendt, M., Rosenberg, R., Foldager, L., Sher, L., & Munk-Jørgensen, P. (2007). Withdrawal symptoms do not predict relapse among subjects treated for cannabis dependence. The American journal on addictions, 16(6), 461–467. https://doi.org/10.1080/10550490701640985
*Back, S. E., Hartwell, K., DeSantis, S. M., Saladin, M., McRae-Clark, A. L., Price, K. L., Moran-Santa Maria, M. M., Baker, N. L., Spratt, E., Kreek, M. J., & Brady, K. T. (2010). Reactivity to laboratory stress provocation predicts relapse to cocaine. Drug and alcohol dependence, 106(1), 21–27. https://doi.org/10.1016/j.drugalcdep.2009.07.016
*Boden, M. T., McKay, J. R., Long, W. R., & Bonn-Miller, M. O. (2013). The effects of cannabis use expectancies on self-initiated cannabis cessation. Addiction (Abingdon, England), 108(9), 1649–1657. https://doi.org/10.1111/add.12233
*Bonn-Miller, M. O., & Moos, R. H. (2009). Marijuana discontinuation, anxiety symptoms, and relapse to marijuana. Addictive behaviors, 34(9), 782–785. https://doi.org/10.1016/j.addbeh.2009.04.009
*Brecht, M. L., & Herbeck, D. (2014). Time to relapse following treatment for methamphetamine use: a long-term perspective on patterns and predictors. Drug and alcohol dependence, 139, 18–25. https://doi.org/10.1016/j.drugalcdep.2014.02.702
*Brecht, M. L., von Mayrhauser, C., & Anglin, M. D. (2000). Predictors of relapse after treatment for methamphetamine use. Journal of psychoactive drugs, 32(2), 211–220. https://doi.org/10.1080/02791072.2000.10400231
*Buydens-Branchey, L., & Branchey, M. (2003). Association between low plasma levels of cholesterol and relapse in cocaine addicts. Psychosomatic medicine, 65(1), 86–91. https://doi.org/10.1097/01.psy.0000039754.23250.ee
*Chen, T., Zhong, N., Du, J., Li, Z., Zhao, Y., Sun, H., Chen, Z., Jiang, H., & Zhao, M. (2019). Polydrug use patterns and their impact on relapse among heroin-dependent patients in Shanghai, China. Addiction (Abingdon, England), 114(2), 259–267. https://doi.org/10.1111/add.14451
*Contreras-Rodríguez, O., Albein-Urios, N., Perales, J. C., Martínez-Gonzalez, J. M., Vilar-López, R., Fernández-Serrano, M. J., Lozano-Rojas, O., & Verdejo-García, A. (2015). Cocaine-specific neuroplasticity in the ventral striatum network is linked to delay discounting and drug relapse. Addiction (Abingdon, England), 110(12), 1953–1962. https://doi.org/10.1111/add.13076
*Domino, K. B., Hornbein, T. F., Polissar, N. L., Renner, G., Johnson, J., Alberti, S., & Hankes, L. (2005).

Risk factors for relapse in health care professionals with substance use disorders. JAMA, 293(12), 1453–1460. https://doi.org/10.1001/jama.293.12.1453

*Fatseas, M., Denis, C., Massida, Z., Verger, M., Franques-Rénéric, P., & Auriacombe, M. (2011). Cue-induced reactivity, cortisol response and substance use outcome in treated heroin dependent individuals. Biological psychiatry, 70(8), 720–727. https://doi.org/10.1016/j.biopsych.2011.05.015

*Fitzpatrick, R. E., Robinson, A. H., Rubenis, A. J., Lubman, D. I., & Verdejo-Garcia, A. (2021). Lack of longitudinal changes in cognition in individuals with methamphetamine use disorder during the first 6 weeks after commencing treatment. The American journal of drug and alcohol abuse, 47(3), 383–392. https://doi.org/10.1080/00952990.2020.1869243

*Flórez-Salamanca, L., Secades-Villa, R., Budney, A. J., García-Rodríguez, O., Wang, S., & Blanco, C. (2013). Probability and predictors of cannabis use disorders relapse: results of the National Epidemiologic Survey on Alcohol and Related Conditions (NESARC). Drug and alcohol dependence, 132(1-2), 127–133. https://doi.org/10.1016/j.drugalcdep.2013.01.013

*Gray, K. M., Sonne, S. C., McClure, E. A., Ghitza, U. E., Matthews, A. G., McRae-Clark, A. L., Carroll, K. M., Potter, J. S., Wiest, K., Mooney, L. J., Hasson, A., Walsh, S. L., Lofwall, M. R., Babalonis, S., Lindblad, R. W., Sparenborg, S., Wahle, A., King, J. S., Baker, N. L., Tomko, R. L., … Levin, F. R. (2017). A randomized placebo-controlled trial of N-acetylcysteine for cannabis use disorder in adults. Drug and alcohol dependence, 177, 249–257. https://doi.org/10.1016/j.drugalcdep.2017.04.020

*Krupitsky, E. M., Zvartau, E. E., Masalov, D. V., Tsoi, M. V., Burakov, A. M., Egorova, V. Y., Didenko, T. Y., Romanova, T. N., Ivanova, E. B., Bespalov, A. Y., Verbitskaya, E. V., Neznanov, N. G., Grinenko, A. Y., O'Brien, C. P., & Woody, G. E. (2004). Naltrexone for heroin dependence treatment in St. Petersburg, Russia. Journal of substance abuse treatment, 26(4), 285–294. https://doi.org/10.1016/j.jsat.2004.02.002

*Marissen, M. A., Franken, I. H., Blanken, P., van den Brink, W., & Hendriks, V. M. (2007). Cue exposure therapy for the treatment of opiate addiction: results of a randomized controlled clinical trial. Psychotherapy and psychosomatics, 76(2), 97–105. https://doi.org/10.1159/000097968

*Morgan, N., Daniels, W., & Subramaney, U. (2019). Smoking heroin with cannabis versus injecting heroin: unexpected impact on treatment outcomes. Harm reduction journal, 16(1), 65. https://doi.org/10.1186/s12954-019-0337-z

*Paltun, S. C., Altunsoy, N., Özdemir, S. D., & Okay, I. T. (2017). Does trait mindfulness level affect quitting cannabis use? A six week follow-up study. Archives of Clinical Psychiatry (São Paulo), 44, 139-144. https://doi.org/10.1590/0101-60830000000139

*Prisciandaro, J. J., Myrick, H., Henderson, S., McRae-Clark, A. L., & Brady, K. T. (2013). Prospective associations between brain activation to cocaine and no-go cues and cocaine relapse. Drug and alcohol dependence, 131(1-2), 44–49.

*Rebgetz, S., Hides, L., Kavanagh, D. J., & Choudhary, A. (2016). Prospective recovery of cannabis use in a psychotic population: A qualitative analysis. Addictive behaviors reports, 4, 31–36.

https://doi.org/10.1016/j.abrep.2016.07.001

*Singh, S. M., Mattoo, S. K., Dutt, A., Chakrabarti, K., Nebhinani, N., Kumar, S., & Basu, D. (2008). Long-term outcome of in-patients with substance use disorders: A study from North India. Indian journal of psychiatry, 50(4), 269–273. https://doi.org/10.4103/0019-5545.44750

*Marhe, R., Waters, A. J., van de Wetering, B. J., & Franken, I. H. (2013). Implicit and explicit drug-related cognitions during detoxification treatment are associated with drug relapse: an ecological momentary assessment study. Journal of consulting and clinical psychology, 81(1), 1–12. https://doi.org/10.1037/a0030754

*Martell, B. A., Mitchell, E., Poling, J., Gonsai, K., & Kosten, T. R. (2005). Vaccine pharmacotherapy for the treatment of cocaine dependence. Biological psychiatry, 58(2), 158–164. https://doi.org/10.1016/j.biopsych.2005.04.032

*Verdejo-Garcia, A., Albein-Urios, N., Martinez-Gonzalez, J. M., Civit, E., de la Torre, R., & Lozano, O. (2014). Decision-making impairment predicts 3-month hair-indexed cocaine relapse. Psychopharmacology, 231(21), 4179–4187. https://doi.org/10.1007/s00213-014-3563-9

*Yonkers, K. A., Forray, A., Nich, C., Carroll, K. M., Hine, C., Merry, B. C., Shaw, H., Shaw, J., & Sofuoglu, M. (2014). Progesterone Reduces Cocaine Use in Postpartum Women with a Cocaine Use Disorder: A Randomized,Double-Blind Study. The lancet. Psychiatry, 1(5), 360–367.

Study name	Authors，year	Drug	Event rate	Person×year	Diagnostic method	Co-morbidities and co-drugs	Gender ratio	Age	Age of first drug use	Duration of drug use (year)
Does trait mindfulness level affect quitting cannabis use? A six week follow-up study	Salih, 2017	cannabis	0.4817	18.86	DSM-IV		female0; male1	27.86		
Withdrawal symptoms do not predict relapse among subjects treated for cannabis dependence	Mikkel, 2007	cannabis	0.67	78	ICD-10		female0.194; male0.806	22.8	11.4	
Postdischarge Cannabis Use and Its Relationship to Cocaine, Alcohol, and Heroin Use: A Prospective Study	Efrat, 2005	cannabis	0.3	568.5	DSM-IV	44% suffer from major depression	female0.34; male0.66	36.9		

Probability and predictors of cannabis use disorders relapse: Results of the National Epidemiologic Survey on Alcohol and Related Conditions (NESARC)	Ludwing, 2013	cannabis	0.0663	8225	DSM-IV		female0.3027; male0.6973	49.5	16.91	5.29
Risk Factors for Relapse in Health Care Professionals With Substance Use Disorders	Karen, 2005	opioid	0.25	2920	DSM-IV		female0.16; male0.84			
The effects of cannabis use expectancies on self-initiated cannabis cessation	Matthew, 2013	cannabis	0.49	8.3	DSM-IV-TR	Almost half met current criteria for an anxiety and/or mood disorder	female0.05; male0.95	50.89		
Smoking heroin with cannabis versus injecting heroin: unexpected impact on treatment outcomes	Nirvana, 2019	heroin	0.774	225	DSM-IV		female0.147; male0.853			0.75

Polydrug use patterns and their impact on relapse among heroin-dependent patients in Shanghai, China	Chen, 2008	heroin	0.592	2515	DSM-IV	Alcohol polydrug users among participants	female0.5; male0.5		
Long-term outcome of in-patients with substance use disorders: A study from North India	Singh, 2008	opioid	0.75	103	ICD-10		No report	39.75	
Association Between Low Plasma Levels of Cholesterol and Relapse in Cocaine Addicts	BUYDENS-BRANCHEY, 2003	cocaine	0.375	38	DSM-III		female0; male1	36.8	6.51
Marijuana discontinuation, anxiety symptoms, and relapse to marijuana	Bonn-Miller, 2009	cannabis	0.3	1288	Axis-I diagnosis	6.2% were diagnosed with post-traumatic stress disorder (PTSD), 8.2% had other anxiety disorders, and 8.8% had	female0; male1	39.27	

						depression.			
Probability and predictors of cannabis use disorders relapse: Results of the National Epidemiologic Survey on Alcohol and Related Conditions (NESARC)	Flórez-Salamanca, 2013	cannabis	0.0663	8460	DSM-IV		female0.3027; male0.6973		
Time to relapse following treatment for methamphetamine use: A long-term perspective on patterns and predictors	Brecht, 2014	amphetamines	0.75	1750	DSM-IV		female0.437; male0.563	29	
A Randomized Placebo-Controlled Trial of N-Acetylcysteine for Cannabis Use Disorder in Adults	Gray, 2017	cannabis	0.2235	75.5	DSM-IV-TR		female0.285; male0.715	30.3	15.1

Decision-making impairment predicts 3-month hair-indexed cocaine relapse	Verdejo-Garcia, 2014	cocaine	0.697	8.25	DSM-IV	The number of cigarettes smoked per month in the non-relapse group was 681.13 ± 372.43, and that in the relapse group was 460.56 ± 309.81	female0.152; male0.848	32.88	20.43	3.7
Implicit and explicit drug-related cognitions during detoxification treatment are associated with drug relapse an ecological momentary assessment study.	Reshmi, 2013	heroin	0.574	1.3	DSM-IV		female0.27; male0.73	40.9	22.3	
Prospective associations between brain activation to cocaine and no-go cues and cocaine relapse	James, 2013	cocaine	0.2	0.57	DSM-IV	The alcohol abuse rate in the relapse group was 0, and 21% in the non-relapse group	female0.167; male0.833	46.96		

Vaccine pharmacotherapy for the treatment of cocaine dependence	Bridget, 2005	cocaine	0.89	8	DSM-IV		female0; male1	40.5	21.6	18.3
Vaccine pharmacotherapy for the treatment of cocaine dependence	Bridget, 2005	cocaine	0.43	8	DSM-IV		female0.25; male0.75	40.6	22.8	13.5
Cue-Induced Reactivity, Cortisol Response and Substance Use Outcome in Treated Heroin Dependent Individuals	Fatseas, 2011	heroin	0.4444	4.5	DSM-IV		female0.4444; male0.5556	37.36		10.42
Cocaine-specific neuroplasticity in the ventral striatum network is linked to delay discounting and drug relapse	Oren， 2015	cocaine	0.45	5	DSM-IV-TR	Nineteen individuals meeting DSM-IV-TR criteria for pathological gambling (PG).	female0.05; male0.95	34.6	22.4	

Reactivity to laboratory stress provocation predicts relapse to cocaine	Sudie，2010	cocaine	0.723	4.399	DSM-IV	79.3% of the participants smoke	female0.4717; male0.5283	38.5	20	13.6
Lack of longitudinal changes in cognition in individuals with methamphetamine use disorder during the first 6 weeks after commencing treatment	Rebecca, 2021	amphetamines	0.8442	8.855	DSM-IV	The number of cigarettes per month in the relapse group was 562.5±241.9; in the non-relapse group was 397.5±260.8	female0.2338; male0.7662	32.04		6.94
The therapeutic effects of methylphenidate and matrix-methylphenidate on addiction severity, craving, relapse and mental health in the methamphetamine use disorder.	Aryan, 2020	amphetamines	0.5	5.5	DSM-IV		female0; male1	31		2.3
Naltrexone for heroin dependence treatment in St. Petersburg, Russia.	Evgeny, 2004	opioid	0.72	26	DSM-IV		female0.28, male0.72	20.7		2.9

Cue Exposure Therapy for the Treatment of Opiate Addiction: Results of a Randomized Controlled Clinical Trial	Marissen, 2007	heroin	0.129	31.75	DSM-IV	female0.11; male0.89	32.1		9.2
Progesterone Reduces Cocaine Use in Postpartum Women with a Cocaine Use Disorder: A Randomized, Double-Blind Study	Kimberly, 2015	cocaine	0.2288	5.25	DSM-IV	female1； male0	32	20	7

Effects of ALANON attendance on family perception of inner-city indigents	Friedemann, 1996	cocaine	0.61	10.5	Family Environment Scale (FES), Family Strategy Effectiveness Assessment (ASF-E), and Family APGAR		female0.1, male0.9	41.5		12
Efficacy of a Cognitive-Behavioral Relapse Prevention Model in the Treatment of Opioid Dependence in Iran: A Randomized Clinical Trial	Leili,2018	opioid	0.52	25	DSM-IV		female0.08; male0.92	38	27.84	10.84

Prospective recovery of cannabis use in a psychotic population	Shane, 2016	cannabis	0.32	5.7	Cannabis Use Track Record in the Past 4 Weeks; Operational Criteria Checklist (OPCRIT)		female0.28, male0.72	25.19		0.25
The effect of self-compassion training on craving and self-efficacy in female patients with methamphetamine dependence: a one-year follow-up	abdoli, 2020	amphetamines	0.75	28	DSM-IV		female1. male0	32.9		
Predictors of relapse after treatment for methamphetamine use	Brecht, 2000	amphetamines	0.36	49	1995-1997 Treated for MA in Los Angeles County Publicly Funded Program		female0.33, male0.67	33.4	20.9	

Predictors of relapse after treatment for methamphetamine use	Brecht, 2000	amphetamines	0.51	147	1995-1997 Treated for MA in Los Angeles County Publicly Funded Program		female0.33, male0.67	33.4	20.9
