# Supplementary material for: Task-switching mechanisms under methamphetamine cravings: sex differences in cued and voluntary task-switching
Source: Front Neurosci. 2024 Oct 30;18:1462157. doi: 10.3389/fnins.2024.1462157 (PMC11557557; doi:10.3389/fnins.2024.1462157)
Supplement: Supplementary file 2 [file Data_Sheet_2.pdf]

药物渴求问卷 DDQ

请您根据自己的实际情况，勾选题项的数字，选择最符合的答案，答案没有对错之分，内容绝对保密。下面 13 个句子是关于您现在对冰毒的一些想法：1=非常不同意、2=比较不同意、3=有点不同意、4=一般、5=有点同意、6=比较同意、7=非常同意

	非常不同意	比较不同意	有点不同意	一般	有点同意	比较同意	非常同意
	1	2	3	4	5	6	7
1.如果现在能享用冰毒会让让我感到很满足							
2.我想现在就想享用冰毒							
3.如果我现在开始享用冰毒,我能及时停止这种想法或者行为							
4.如果现在能享用冰毒,我可以做任何事							
5.如果我能享用冰毒,我压根儿都不担心自己的日常生活问题							
6.现在就想享用冰毒的欲望,完全没办法阻挡							
7.如果现在享用冰毒,我很容易就可以限 制自己使用的量							
8.如果我享用冰毒,我感到生活中所有糟 糕的事情全都消失了							
9.我非常想要冰毒就像我已经尝到了它的味道一样							
10.现在享用冰毒能使我感到轻松							
11.一旦我享用冰毒,生活中再大的问题都与我无关							
12.马上享用冰毒能让我愉快							
13.我现在想尽快地享用冰毒							
14.如果现在能享用冰毒会让我感到很满足							
