# Supplementary material for: Task-switching mechanisms under methamphetamine cravings: sex differences in cued and voluntary task-switching
Source: Front Neurosci. 2024 Oct 30;18:1462157. doi: 10.3389/fnins.2024.1462157 (PMC11557557; doi:10.3389/fnins.2024.1462157)
Supplement: Supplementary file 3 [file Data_Sheet_3.pdf]

ANOVA for all 32 fNIRS channels

Channel	Volun-female (M ± SD)	Volun-male (M ± SD)	Cued-female (M ± SD)	Cued-male (M ± SD)	Type F	Type p	Type ηp2	Sex F	Sex p	Sex ηp2	Inter F	Inter p	Inter ηp2
1	-5.420E-04 ± 1.630E-03	1.320E-04 ± 3.210E-04	-7.100E-04 ± 2.329E-03	-1.630E-04 ± 4.230E-04	2.596	0.125	0.126	0.937	0.346	0.049	0.159	0.664	0.011
2	-3.798E-07 ± 1.770E-04	1.220E-04 ± 3.040E-04	1.950E-04 ± 2.170E-04	-7.000E-05 ± 3.370E-04	<0.001	0.985	<0.001	0.704	0.412	0.038	5.383	0.032	0.230
3	3.370E-04 ± 3.318E-03	3.900E-05 ± 3.900E-05	2.662E-03 ± 8.298E-03	-3.050E-04 ± 9.850E-04	0.858	0.367	0.045	1.280	0.273	0.066	1238	0.281	0.064
4	1.149E-03 ± 5.658E-03	-1.978E-03 ± 4.093E-03	-3.877E-03 ± 9.731E-03	-2.442E-03 ± 6.751E-03	1.216	0.285	0.063	0.220	0.645	0.012	0.840	0.372	0.045
5	4.570E-04 ± 2.286E-03	2.630E-04 ± 1.778E-03	-2.315E-03 ± 7.073E-03	2.600E-04 ± 1.241E-03	0.822	0.377	0.044	2.157	0.159	0.107	0.819	0.377	0.044
6	-3.700E-05 ± 1.690E-04	3.590E-04 ± 9.220E-04	-3.220E-04 ± 1.187E-03	1.187E-03 ± 2.253E-03	0.556	0.466	0.030	0.729	0.404	0.039	0.015	0.903	0.001
7	-2.600E-05 ± -	2.900E-05 ±	1.300E-04 ±	-1.383E-03 ±	1.054	0.318	0.055	1.751	0.202	0.089	1.643	0.216	0.084

	2.600E-05	3.210E-04	1.700E-04	3.659E-03									
8	9.422E-06 ± 4.643E-04	2.714E-03 ± 6.451E-03	-1.123E-03 ± 1.890E-03	5.258E-03 ± 1.907E-02	0.110	0.744	0.006	1.296	0.270	0.067	0.745	0.399	0.040
9	1.675E-05 ± 1.091E-04	-9.488E-04 ± 2.890E-03	-6.918E-05 ± 2.815E-04	-4.638E-04 ± 3.039E-03	1.426	0.248	0.073	0.540	0.472	0.029	2.919	0.105	0.140
10	-3.703E-05 ± 1.736E-04	-1.775E-04 ± 1.174E-03	-1.638E-04 ± 2.443E-04	-2.843E-04 ± 9.312E-04	0.157	0.697	0.009	0.574	0.459	0.031	0.001	0.973	<0.001
11	-9.490E-05 ± 3.772E-04	1.850E-03 ± 4.448E-03	3.282E-03 ± 9.821E-03	-2.175E-03 ± 6.087E-03	0.020	0.889	0.001	1.256	0.277	0.065	2.626	0.123	0.127
12	2.791E-04 ± 4.776E-04	-2.384E-03 ± 6.133E-03	2.406E-04 ± 6.512E-04	-4.915E-03 ± 9.527E-03	1.433	0.247	0.074	2.884	0.107	0.138	1.349	0.261	0.070
13	4.634E-04 ± 2.462E-03	2.720E-03 ± 8.020E-03	1.050E-04 ± 5.576E-04	3.087E-03 ± 5.826E-03	<0.001	0.998	<0.001	2.146	0.160	0.107	0.065	0.802	0.004
14	-1.229E-04 ± 3.903E-04	-1.925E-04 ± 5.163E-04	-9.031E-05 ± 4.813E-04	7.016E-05 ± 4.464E-04	1.431	0.247	0.074	0.076	0.768	0.004	0.869	0.364	0,046
15	-8.917E-06 ±	-4.412E-04	-1.041E-04 ±	-2.176E-04 ±	0.385	0.543	0.021	5.466	0.031	0.233	2.369	0.141	0.116

	2.377E-04	± 4.874E-04	2.701E-04	3.465E-04									
16	-5.335E-05 ±	-1.765E-04	3.636E-06 ±	-9.730E-05 ±	0.528	0.477	0.028	1.060	0.317	0.056	0.014	0.907	0.001
	1.432E-04	± 4.928E-04	2.934E-04	2.511E-04									
17	-5.498E-05 ±	2.546E-04 ±	-1.009E-04 ±	1.469E-04 ±	0.338	0.568	0.018	4.535	0.047	0.201	0.055	0.818	0.003
	1.408E-04	6.337E-04	2.808E-04	4.374E-04									
18	-2.173E-05 ± -	1.578E-04 ±	-4.758E-04 ±	-2.806E-04 ±	3.555	0.076	0.165	0.789	0.386	0.042	0.001	0.974	<0.001
	2.173E-05	3.246E-04	1.042E-03	8.689E-04									
19	-4.020E-06 ±	2.124E-04 ±	5.512E-05 ±	2.414E-03 ±	1.634	0.217	0.083	0.950	0.343	0.050	1.467	0.241	0.075
	3.999E-04	1.776E-03	6.119E-04	6.846E-03									
20	-8.877E-05 ±	3.033E-03 ±	7.021E-05 ±	2.378E-03 ±	0.304	0.588	0.017	1.130	0.302	0.059	0.819	0.377	0.044
	5.575E-04	9.305E-03	5.341E-04	6.878E-03									
21	-1.410E-04 ±	4.227E-04 ±	-1.078E-04 ±	3.060E-04 ±	0.083	0.776	0.005	1.758	0.201	0.089	0.268	0.611	0.015
	2.881E-04	1.420E-03	4.459E-04	9.158E-04									
22	5.645E-04 ±	-1.098E-03	-4.776E-04 ±	1.937E-03 ±	0.375	0.548	0.020	0.169	0.686	0.009	1.568	0.227	0.080
	1.902E-03	± 3.534E-03	4.690E-03	5.629E-03									
23	6.818E-05 ±	2.367E-03 ±	-1.781E-05 ±	1.395E-04 ±	1.041	0.321	0.055	0.853	0.368	0.045	0.892	0.357	0.047

	4.248E-04	7.673E-03	1.083E-03	9.288E-04									
24	-1.740E-03 ± 4.933E-03	-9.082E-05 ± 2.883E-04	2.941E-03 ± 8.517E-03	-1.382E-04 ± 4.762E-04	1.187	0.290	0.062	1.524	0.233	0.078	1.236	0.281	0.064
25	-8.344E-05 ± 2.387E-04	-2.675E-04 ± 4.760E-04	2.769E-05 ± 6.857E-04	-1.331E-04 ± 5.138E-04	0.781	0.389	0.042	0.942	0.345	0.050	0.007	0.934	<0.001
26	-1.901E-04 ± 3.882E-04	-1.160E-04 ± 4.030E-04	-6.874E-05 ± 4.352E-04	-1.573E-04 ± 4.417E-04	0.072	0.791	0.004	0.004	0.949	<0.001	0.299	0.591	0.016
27	3.633E-04 ± 7.098E-04	-1.243E-06 ± 3.992E-04	3.918E-04 ± 9.544E-04	-1.212E-03 ± 4.078E-03	0.685	0.419	0.037	2.420	0.137	0.118	0.753	0.397	0.040
28	4.315E-04 ± 8.535E-04	1.017E-05 ± 2.367E-04	4.339E-04 ± 2.833E-04	2.644E-05 ± 3.421E-04	0.004	0.949	<0.001	6.076	0.024	0.252	0.002	0.962	<0.001
29	-8.343E-04 ± 1.658E-03	7.085E-04 ± 3.528E-03	6.775E-04 ± 1.815E-03	5.438E-04 ± 2.195E-03	1.338	0.263	0.069	0.601	0.448	0.032	2.072	0.167	0.103
30	-6.675E-04 ± 1.976E-03	-7.469E-04 ± 1.587E-03	4.312E-04 ± 1.181E-03	-2.777E-04 ± 1.234E-03	0.142	0.093	0.149	0.573	0.459	0.031	0.506	0.486	0.027
31	-6.449E-06 ±	-1.596E-04	3.769E-05 ±	1.711E-04 ±	1.097	0.309	0.057	0.013	0.912	0.001	0.641	0.434	0.034

	1.708E-04	± 5.165E-04	1.810E-04	6.827E-04									
32	2.218E-05 ±	1.470E-04 ±	1.378E-05 ±	1.845E-04 ±	0.020	0.888	0.001	0.943	0.344	0.050	0.051	0.824	0.003
	2.159E-04	4.304E-04	3.039E-04	5.872E-04									
